# Seminal citrate is superior to PSA for detecting clinically significant prostate cancer

**DOI:** 10.1590/S1677-5538.IBJU.2018.0730

**Published:** 2019-12-17

**Authors:** Emerson Pereira Gregório, Antonio Paulo Alexandrino, Ivania Terezinha Albrecht Schuquel, Willian Ferreira da Costa, Marco Aurelio de Freitas Rodrigues

**Affiliations:** 1 Pontifícia Universidade Católica do Paraná (PUCPR), Faculdade de Medicina, Londrina, PR, Brasil; 2 Disciplina de Urologia, Departamento de Cirurgia Clínica, Universidade Estadual de Londrina (UEL), Londrina, PR, Brasil; 3 Departamento de Química, Universidade Estadual de Maringá (Universidade Estadual de Maringá-UEM), Maringá, PR, Brasil

**Keywords:** Prostate-Specific Antigen, Prostate cancer, familial [Supplementary Concept], Citrates

## Abstract

**Purpose:**

To establish whether the citrate concentration in the seminal fluid ([CITRATE]) measured by means of high-resolution nuclear magnetic resonance spectroscopy (1HNMRS) is superior to the serum prostate-specific antigen (PSA) concentration in detecting of clinically significant prostate cancer (csPCa) in men with persistently elevated PSA.

**Materials and Methods:**

The group of patients consisted of 31 consecutively seen men with histological diagnosis of clinically localized csPCa. The control group consisted of 28 men under long-term follow-up (mean of 8.7 ± 3.0 years) for benign prostate hyperplasia (BPH), with persistently elevated PSA (above 4 ng/mL) and several prostate biopsies negative for cancer (mean of 2.7 ± 1.3 biopsies per control). Samples of blood and seminal fluid (by masturbation) for measurement of PSA and citrate concentration, respectively, were collected from patients and controls. Citrate concentration in the seminal fluid ([CITRATE]) was determined by means of 1HNMRS. The capacities of PSA and [CITRATE] to predict csPCa were compared by means of univariate analysis and receiver operating characteristic (ROC) curves.

**Results:**

Median [CITRATE] was significantly lower among patients with csPCa compared to controls (3.93 mM/l vs. 15.53 mM/l). There was no significant difference in mean PSA between patients and controls (9.42 ng/mL vs. 8.57 ng/mL). The accuracy of [CITRATE] for detecting csPCa was significantly superior compared to PSA (74.8% vs. 54.8%).

**Conclusion:**

Measurement of [CITRATE] by means of 1HNMRS is superior to PSA for early detection of csPCa in men with elevated PSA.

## INTRODUCTION

The practice of performing systematic biopsies in all men with elevated serum total prostate-specific antigen (PSA) levels causes unacceptable rates of over diagnosis and overtreatment of clinically insignificant prostate cancer (1). In recent years, the volume of prostate tumors detected by means of PSA has decreased, consequently, PSA has been more correlated with prostate gland volume than with tumor volume (2). For this reason, a large population of men with persistently elevated PSA and one or more negative prostate biopsies are now at risk of developing clinically significant prostate cancer (csPCa) (3, 4).

These facts, in addition to the financial cost, morbidity risk and emotional problems associated with repeated prostate biopsies, point to the need for developing noninvasive and more accurate alternatives to the PSA-digital rectal exam (DRE) combination for prostate cancer (PCa) detection.

Most investigators seeking such alternatives have focused on identifying plasma tumors or genetic markers. However, recent studies point to a possible marker in the seminal fluid (5, 6).

The aim of the present study was to establish whether the citrate concentration in the seminal fluid ([CITRATE]) is superior to PSA for detection csPCa in men with elevated PSA.

## MATERIAL AND METHODS

Trial design and participants

**Figure 1:**
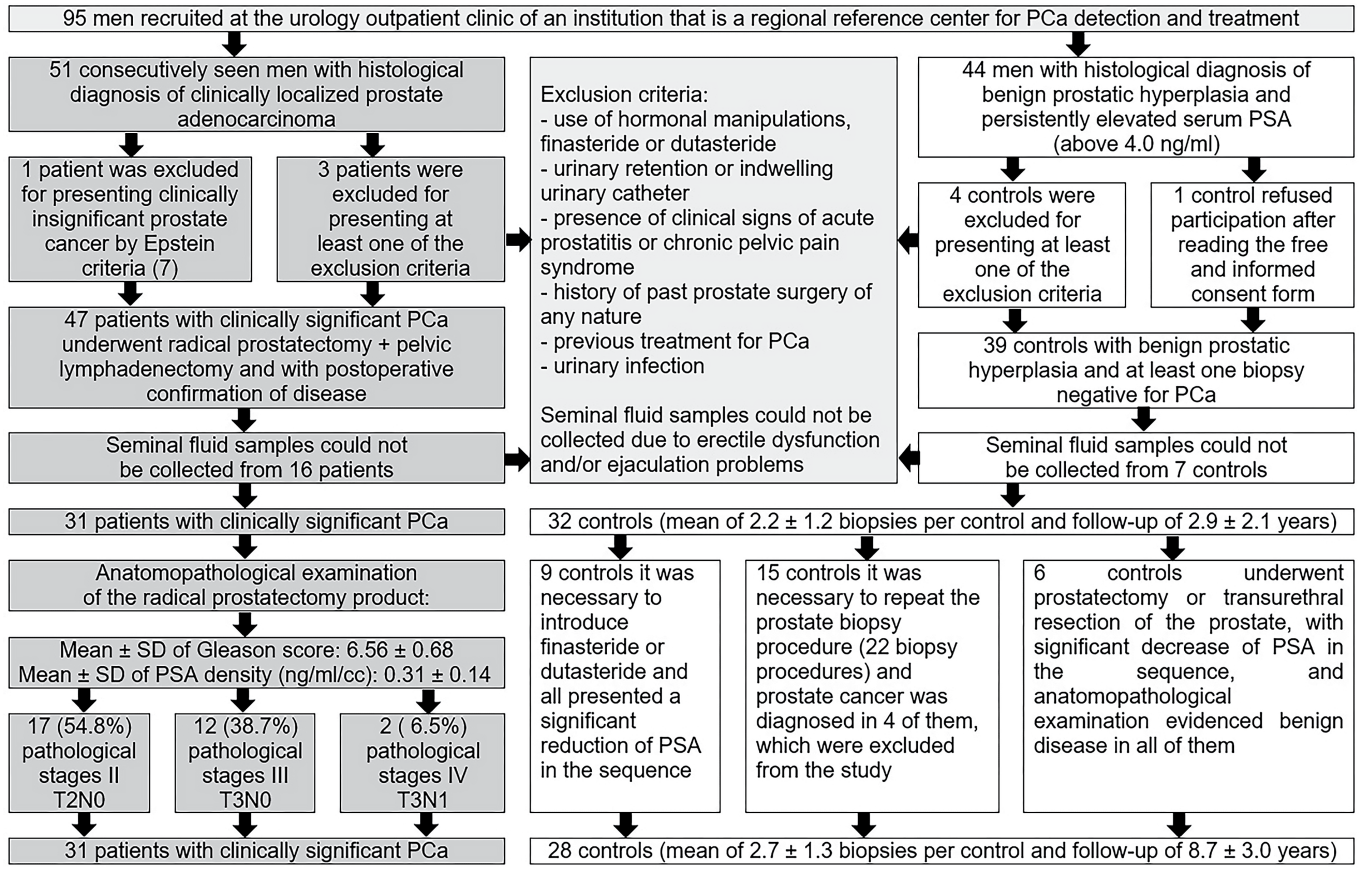
Distribution of patients and controls and the flowchart of exclusion and follow-up of the participants.

This study was conducted to assess a diagnostic test and was approved by the Ethics Committee of the institution. All participants signed a free and informed consent form. The distribution of patients and controls and the flowchart of exclusion and follow-up of the participants are shown in Figure-1. The Epstein criteria were applied for determining whether prostate cancer was clinically insignificant (7).

### Measurement of PSA and [CITRATE]

Blood samples were collected from patients and control immediately before collection of seminal fluid and any manipulation of the prostate for measurement of PSA levels. PSA was measured using a chemiluminescence assay (ADVIA Centaur CP, Siemens Healthcare Diagnostics Inc., Tarrytown, NY 10591-5097 USA) according to the manufacturer's instructions.

Semen samples for measurement of [CITRATE] were collected from patients and controls by means of masturbation. For this purpose, the participants were requested to abstain from ejaculating for at least three days, sample collection was performed at least six weeks after prostate biopsy. Those men who had made previous use of oral phosphodiesterase type 5 inhibitors to achieve or sustain erection were instructed to use the medication one hour before sample collection.

The volume of the seminal fluid samples was measured, and the samples were then pipetted in 0.5mL aliquots into cryogenic vials sealed within polypropylene tube packages and stored in liquid nitrogen tanks. The samples were then lyophilized using a vacuum lyophilizer (Labiconc lyophilizer, with a 1kg ice capacity and a V8 vacuum pump, Edwards do Brasil) and were stored in a refrigerator at approximately 4°C until submitted to high-resolution magnetic nuclear resonance spectroscopy (1HNMRS).

To measure [CITRATE], a calibration curve was elaborated using standard citrate solutions with TSP (3-trimethylsilylpropionic-2, 2, 3, 3d acid sodium salt) as the internal standard. Nine standard solutions of citrate in heavy water (D2O) were prepared at concentrations of 4, 20, 40, 60, 80, 100, 120, 160 and 200mM in the presence of 5.93mM TSP. Aliquots of 0.5mL of each such concentrations were separately pipetted into 5mm nuclear magnetic resonance (NMR) tubes, and the high-resolution nuclear magnetic resonance (1HNMR) spectra were immediately acquired. The calibration curve was obtained by plotting the ratio of the relative area of citrate to the relative area of the internal standard, both obtained in the 1HNMR spectrum as a function of the citrate concentration.

Immediately before the analyses, the lyophilized seminal fluid samples were redissolved in 1mL of 5.93mM TSP in D2O and transferred to 5mm NMR tubes. The 1HNMR spectra were obtained using a Varian Mercury plus BB spectrometer at 300.059MHz for 1H equipped with a 5mm direct detection probe with field gradient, at room temperature (∼23°C) and referenced relative to TSP (δ 0.00ppm). In total, 64 flow induction decays (FIDs) with 28.450 data points (np) and 3.908Hz of spectral width (sw) were collected from each sample, with 45° pulses and a recycle time (d1) of 10s. To improve the signal/noise ratio, an exponential apodization function was applied to the flow induction decay (FID), resulting in line broadening (lb) of 1Hz. Next, the baseline of the spectra was corrected, and the resonance signals of citrate (four lines) and TSP were integrated.

### Minimal risk of occult PCa among controls

The controls were followed every six months, with DRE, PSA, free PSA, calculation of percent free PSA, calculation of the speed of PSA rise, calculation of PSA density using the prostate volume measured in previous prostate biopsy. Biopsy of at least 12 prostate fragments was performed in controls with suspicious abnormalities in the aforementioned tests. Biopsy was repeated in controls with atypical small acinar proliferation (ASAP), controls with prostatic intraepithelial neoplasia (PIN) and controls in whom the previously mentioned abnormalities persist.

Until seminal fluid collection, the control group consisted of 32 men under long-term follow-up (mean of 2.9±2.2 years) for benign prostate hyperplasia (BPH), with persistently elevated PSA and several prostate biopsies negative for cancer (mean of 2.2±1.2 biopsies per control). Due to the risk of occult PCa, after collection of seminal fluid, these 32 controls continued to be prospectively followed-up at the outpatient clinic according to the protocol described above, for a mean time of 6.2±2.0 years. In 15 controls it was necessary to repeat the prostate biopsy (22 biopsy procedures) and PCa was diagnosed in four of them, which were excluded from the study. In nine controls it was necessary to introduce finasteride or dutasteride and all presented a significant reduction of PSA. Six controls underwent prostatectomy or transurethral resection of the prostate, with significant decrease of PSA as well, and anatomopathological examination evidenced benign disease in all of them (Figure-1).

At the end of the study, the sample size consisted of 31 patients ranging from 47 to 73 years old and serum PSA levels ranging from 3.67 to 17.50ng/mL. The control group consisted of 28 men ranging from 51 to 75 years old and PSA levels ranging from 4.15 to 15.50ng/mL. Mean follow-up for the control group was 8.7 years with several prostate biopsies negative for PCa (mean of 2.7±1.3 biopsies per control). The characteristics of the 28 controls are described in Table-1.

**Table 1:** Characteristics of the 28 controls included in the study.

Characteristics of the 28 controls	Mean ± standard deviation
Length of follow-up (years)	8.7±3.0
Number of prostate biopsies per control	2.7±1.3
Serum PSA (ng/mL)	8.57±3.17
Percent free PSA (%)	17.09±5.62
Prostate volume (cc)	68.13±26.71
PSA density (ng/mL/cc)	0.14±0.07

Positive linear correlation was identified between serum PSA and number of prostate biopsies in the 28 controls (correlation coefficient: 0.534; p= 0.005)

#### Statistical analysis

A significance level of p <0.05 and a 95% confidence interval (CI) were adopted in all analyses. Analysis was performed using the statistical software Medcalc for Windows version 9.5.2.0 (Medcalc Software, Mariakerke, Belgium).

The required sample size was calculated from a similar study (5) which measured [CITRATE] by 1HNMRS in 21 patients with PCa and 16 controls and found an area under the ROC curve (AUC) of [CITRATE] for PCa detection of 0.81. For α-level of 0.05 and for β-level of 0.20 (statistical power of 80%), the sample size calculated was 27 controls and 27 patients.

The T-test (when accepted to normality) and the Mann-Whitney U test (when rejected normality) were used to compare means and medians, respectively, between groups. The capacity of PSA and [CITRATE] to predict csPCa was assessed by means of sensitivity, specificity and ROC (receiver operating characteristic) curves. The AUC were compared as described by Hanley & McNeil (1983) (8).

## RESULTS

The characteristics of patients and controls and the comparison between groups (csPCa and BPH) are described in Table-2. [CITRATE] was significantly lower in the patient group compared to the controls. There were no differences between the groups relative to the remaining variables (age, serum PSA and volume of seminal fluid).

**Table 2:** Comparison of age (years), PSA (ng/mL), seminal fluid volume (SF) (mL) and [CITRATE] (mm/l) in men with BPH and csPCa.

Variable	BPH 28 controls (47%) Mean (SD)	csPCa 31 patients (53%) Mean (SD)	p value
Age (years)	64.46	63.39	0.5392*
	(7.02)	(6.38)	
PSA (ng/mL)	8.57	9.42	0.3623*
	(3.17)	(3.85)	
SF volume (mL)	1.52	1.35	0.5402*
	(0.98)	(1.12)	
[CITRATE] (mM/L)	24.58	7.60	0.0011**
	(22.17)	(10.22)	
	Median = 15.53	Median = 3.93	
	IR = 5.71-39.71	IR = 1.38-10.74	

* T-test

** Mann-Whitney U test

SD = Standard Deviation

IR = Interquartile Range

**Figure 2:**
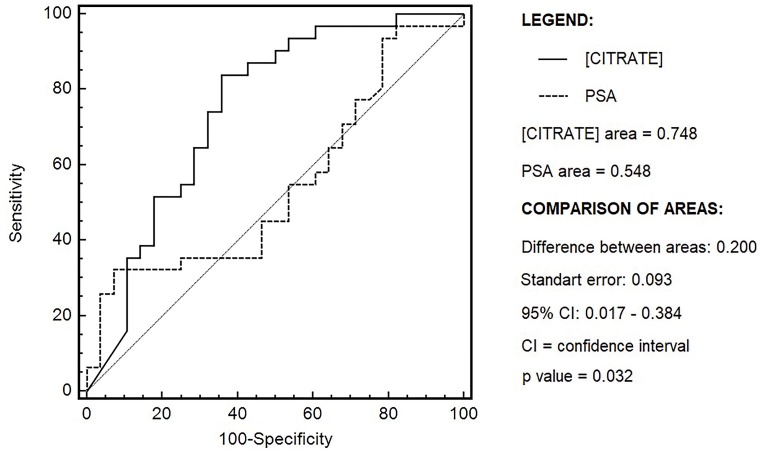
ROC curves corresponding to variables [CITRATE] and PSA and comparison of the areas under the curve.

Figure-2 depicts the ROC curves corresponding to variables PSA and [CITRATE] and the comparison of the AUC. The AUC of [CITRATE] to detect csPCa was superior compared to PSA (p=0.032).

The cutoff points and specificity of [CITRATE] and PSA to detect csPCa relative to arbitrary values of sensitivity are described in Table-3.

**Table 3:** Specificity and cutoff points of [CITRATE] and PSA for arbitrary values of sensitivity relative to detection of csPCa.

% Sensitivity % detected csPCa	[CITRATE] (mM/l) cutoff point	% [CITRATE] Specificity	PSA (ng/mL) cutoff point	% PSA Specificity
95.0	29.75	39.29	4.21	17.86
90.0	14.75	50.00	4.55	21.43
75.0	9.92	64.29	6.55	28.57
50.0	3.93	82.14	7.87	46.43
25.0	2.27	89.29	13.05	96.43

## DISCUSSION

Under normal conditions, the glandular epithelial cells in the peripheral region of the human prostate are able to accumulate large amounts of zinc and to synthesize, store and secrete extraordinarily large amounts of citrate. The hyperplastic prostate (BPH) is also able to accumulate zinc and to produce citrate. The development of PCa necessarily involves metabolic changes, whereby the malignant cells become unable to accumulate zinc and citrate. Parallel to the reduction in the intracellular citrate concentration, the citrate levels decrease in the prostatic fluid and, consequently, in the seminal fluid (9-18).

The normal value of the citrate concentration in the seminal fluid ([CITRATE]) depends on the methodology used for measurement. A study (19) that evaluated 30 healthy young men (mean age 38.03±10.06 years), using a methodology identical to that of the current study to measure [CITRATE], found a median of [CITRATE] in these men of 44.68mM/l. In the current study, median [CITRATE] was 3.95-fold higher in controls with BPH than in patients with PCa (15.53 vs. 3.93mM/l, p=0.0011).

The inability of PCa cells to accumulate zinc and to produce citrate, and the capacities of the cells of the hyperplastic prostate (BPH) to accumulate zinc and to produce, store and secrete citrate are the bases for the advantage of [CITRATE] over PSA as a diagnostic test for PCa detection. The reason for this advantage is that PSA is elevated in both PCa and BPH, while [CITRATE] is decreased in PCa, remaining elevated in BPH only (14).

Taking those facts into consideration, BPH represents a potential cause of false-negative results in the use of [CITRATE] for the detection of PCa (high [CITRATE] in the presence of PCa). However, the increase of the citrate concentration in the central zone of the hyperplastic prostate is not sufficient to “mask” the decrease of the citrate concentration in the malignant cells in the peripheral zone of the gland. According to the currently available evidence, the prostatic fluid, even in the presence of BPH, reflects the metabolic conditions of the peripheral zone of the prostate (15).

The aforementioned characteristics of the marker measured in the seminal fluid account for the results of both this and another study (5), in which the median [CITRATE] was lower in the patients with PCa compared to the controls and the AUC of [CITRATE] to detect PCa was superior compared to PSA. In the present study, the respective AUC of [CITRATE] and PSA to detect csPCa were 0.748 and 0.548. In Kline et al. (5), the respective AUC of [CITRATE] and PSA to detect PCa were 0.81 and 0.61.

In addition to the larger sample size, another advantage of the present study compared to Kline et al. (5) is that all of the participants in the control group had negative biopsies for PCa (2.7±1.3 biopsies per control) and were followed up prospectively (8.7±3.0 years), before and after collection of the seminal fluid, with assessment of the speed of PSA rise, percent free PSA (%fPSA), PSA density and additional biopsies as needed, thus minimizing the risk of false-negative biopsy results (occult PCa).

In Kline et al. (5), the control group included young men under 30 years old and men with PSA above 4ng/mL who had never been submitted to biopsy. These characteristics of the controls partially account for the greater AUC of PSA to detect PCa (0.61) compared to the present study (0.548). Another study (3), which assessed diagnostic tests for PCa detection, in which the characteristics of the control group were similar to the characteristics in the present study, found that the AUC of PSA to detect PCa was 0.524, and thus, quite close to the AUC measured in the present study (0.548).

Currently, there are numerous options to improve early detection as compared to a purely prostate-specific antigen (PSA)-based approach. All have strengths and drawbacks. In addition to repeating the PSA and performing clinical work-up (digital rectal examination and estimation of prostate volume), additional tests investigated in the initial biopsy setting are: %fPSA, Prostate Health Index (Phi), 4-kallikrein score (4KScore), SelectMDx, and Michigan Prostate Score and in the repeat setting: %fPSA, Phi, 4KScore, Prostate Cancer Antigen 3 (PCA3), and ConfirmMDx (20). With the exception of %fPSA, all these biomarkers are costly and are scarcely available in our country.

The 2016 National Comprehensive Cancer Network (NCCN) guideline recommends any of the following reflex tests (blood) to follow an elevated PSA >3ng/mL: %fPSA, 4K Score or Phi (21). Recent diagnostic accuracy studies assessing a %fPSA (22), 4KScore (23) and Phi (22) showed an AUC of 0.63, 0.72 and 0.74, respectively, for PCa detection. Those values are lower than the ones of the AUC of [CITRATE] for csPCa detection measured in the present study (0.748).

Although there have been a multitude of potential biomarkers that in preliminary studies were proven to be better than PSA, there are few studies of diagnostic accuracy for csPCa detection that evaluated men with persistently elevated PSA and several prostate biopsies negative for PCa. A study (3), which assessed diagnostic tests for PCa detection (any grade), in which the characteristics of the control group were similar to the characteristics in the present study, found that the AUC of PCA3 to detect PCa was 0.68, therefore, lower than that of [CITRATE].

Available results about the PCA3 showed its usefulness to decide the repetition of biopsy in patients with a previous negative result, although its relationship with the aggressiveness of the tumor is controversial. On the other hand, recent diagnostic accuracy studies assessing a 4K Score and Phi showed an AUC ranging from 0.71 to 0.74 for high-grade PCa detection (24, 25). Those values are similar to the ones of the AUC of [CITRATE] for csPCa detection measured in the present study (0.748).

The use of Prostate Imaging Reporting and Data System Version 2 (PI-R ADS-v2) with multiparametric magnetic resonance imaging (mpMRI) for the detection of PCa appears to have good diagnostic accuracy in patients with PCa lesions with high sensitivity (0.85) and moderate specificity (0.71) (26). However, the decision of whether to perform PI-RADS-v2 in this setting must also take into account the results of biomarkers, cost, as well as the availability of high quality mpMRI interpretation (27).

Therefore, there are numerous tests available that can help increase the specificity of PSA, in the initial and repeat biopsy setting, all coincident with a small decrease in sensitivity of detecting high-grade cancer. PI-RADS-v2 with mpMRI is an important diagnostic adjunct. Cost effectiveness is crucial. The way forward is a multivariable risk assessment on the basis of readily available clinical data, potentially with the addition of PSA subforms, preferably at low cost (20).

The cost, in Canadian dollars, of measuring [CITRATE] is only $50.00 (28). On the other hand, the cost, in Canadian dollars, of Phi, PCA3 and 4K Score are higher, respectively, $150.00, $385.00 and $800.00 (29). In this way, due to the low cost and good accuracy of [CITRATE] for csPCa detection, it could be included this new biomarker to be used for the multivariable risk assessment in the initial and repeat biopsy setting or to select patients who will perform the PI-RADS-v2 with mpMRI. Future studies comparing accuracy of [CITRATE] with that of PI-RADS-v2 for detecting csPCa should also be encouraged. Measurement of [CITRATE] may also be evaluated in future studies as a prognostic biomarker of PCa.

Compared to serum PSA, measurement of [CITRATE] is disadvantageous relative to the practical aspects of sample collection and cannot be used in the follow-up of patients with PCa subjected to radical prostatectomy or of men with BPH subjected to surgery who subsequently develop retrograde ejaculation.

Erectile dysfunction and/or ejaculation problems further make the collection of seminal fluid difficult. In the present study, samples could not be collected through masturbation in 34% of the patients and 18% of the controls. In such cases, the samples might be collected following transrectal massage of the prostate. Kline et al. (5) observed a non-significant diagnostic difference between using citrate concentration from seminal fluid or prostatic fluid for PCa detection. In the present study, collection of seminal fluid through masturbation was preferred due to the difficulties involved in the collection of prostatic fluid following transrectal massage of the prostate. Some authors consider (6) that the use of prostatic fluid as a tumor marker for PCa detection might be unfeasible as a function of the need of strong transrectal massage of the prostate to collect the biological material, which might cause discomfort to the men, and thus might not be tolerated as a routine test. To minimize such discomfort, an alternative might be to collect prostatic fluid through transrectal massage with the patient under sedation immediately before a surgical biopsy indicated due to elevated PSA or abnormal findings on DRE. In such cases, the citrate concentration in the prostatic fluid might serve as a further criterion to reinforce the need to repeat the prostate biopsy.

Considering the importance of the early diagnosis of cases of potentially curable PCa, the use of cutoff points resulting in high sensitivity without any loss of specificity is interesting. In the present study, for the cutoff points associated with high rates of sensitivity, the specificity rates of [CITRATE] were more than twice as high compared to PSA, resulting in a reduction of the number of unnecessary prostate biopsies by at least 50%. The specificities of both tests were similar at the lower levels of sensitivity, however, such levels are not interesting in actual practice because many cases of potentially curable PCa will not be diagnosed (Table-3).

The introduction of new tumor markers in clinical practice is complex and includes several stages, i.e., transition from the stage of discovery to pre-validation in retrospective and prospective studies, validation in multicenter studies, approval by regulatory agencies, and finally commercialization and release to the final users (30).

## CONCLUSIONS

Mensuration of [CITRATE] by means of 1HNMRS is superior to PSA for detection of csPCa in men with elevated PSA. However, multicenter and prospective studies with larger samples, are needed.
